# Dynamic Imaging of Coherent Sources Reveals Different Network Connectivity Underlying the Generation and Perpetuation of Epileptic Seizures

**DOI:** 10.1371/journal.pone.0078422

**Published:** 2013-10-23

**Authors:** Lydia Elshoff, Muthuraman Muthuraman, Abdul Rauf Anwar, Günther Deuschl, Ulrich Stephani, Jan Raethjen, Michael Siniatchkin

**Affiliations:** 1 Department of Neuropediatrics, Christian-Albrechts-University, Kiel, Germany; 2 Department of Neurology, Christian-Albrechts-University, Kiel, Germany; 3 Digital Signal Processing and System Theory, Technical Faculty, Christian-Albrechts-University, Kiel, Germany; 4 Department of Child and Adolescent Psychiatry, Psychosomatics and Psychotherapy, Goethe-University of Frankfurt am Main, Frankfurt, Germany; Medical University of Vienna, Austria

## Abstract

The concept of focal epilepsies includes a seizure origin in brain regions with hyper synchronous activity (epileptogenic zone and seizure onset zone) and a complex epileptic network of different brain areas involved in the generation, propagation, and modulation of seizures. The purpose of this work was to study functional and effective connectivity between regions involved in networks of epileptic seizures. The beginning and middle part of focal seizures from ictal surface EEG data were analyzed using dynamic imaging of coherent sources (DICS), an inverse solution in the frequency domain which describes neuronal networks and coherences of oscillatory brain activities. The information flow (effective connectivity) between coherent sources was investigated using the renormalized partial directed coherence (RPDC) method. In 8/11 patients, the first and second source of epileptic activity as found by DICS were concordant with the operative resection site; these patients became seizure free after epilepsy surgery. In the remaining 3 patients, the results of DICS / RPDC calculations and the resection site were discordant; these patients had a poorer post-operative outcome. The first sources as found by DICS were located predominantly in cortical structures; subsequent sources included some subcortical structures: thalamus, Nucl. Subthalamicus and cerebellum. DICS seems to be a powerful tool to define the seizure onset zone and the epileptic networks involved. Seizure generation seems to be related to the propagation of epileptic activity from the primary source in the seizure onset zone, and maintenance of seizures is attributed to the perpetuation of epileptic activity between nodes in the epileptic network. Despite of these promising results, this proof of principle study needs further confirmation prior to the use of the described methods in the clinical praxis.

## Introduction

An epileptic seizure exists when neurons in the cerebral cortex are excessively and hyper-synchronously activated. Focal epileptic seizures result from a circumscribed region of hyper-synchronous activity called the seizure onset zone (SOZ) and may propagate to other regions in the brain [1]. Knowledge about the SOZ in a particular patient is essential for treatment strategies such as epilepsy surgery or brain stimulation. However, the neuronal networks involved in the generation and maintenance of epileptic seizures may be more complex than only an SOZ. Epileptic networks may include structures which 1) are involved in the propagation of epileptic activity from the SOZ, 2) exert a modulatory influence on the SOZ by increasing or decreasing pathological synchronization (e.g., thalamo-cortical network), 3) or represent a reactivity of remote functional brain regions on epileptic activity (e.g., default mode network) [2-4]. The precise characterization of both the SOZ as well as the entire epileptic network may improve therapeutic options for epilepsies and our understanding of mechanisms and functional consequences of epileptic seizures.

At present, different methods have been developed to accurately detect the SOZ as well as the associated epileptic networks. Most of them are related to functional brain imaging such as positron emission tomography (PET), ictal single photon emission computed tomography (ictal SPECT), and functional magnetic resonance imaging (fMRI) with and without simultaneous EEG (for review 5). None of these diagnostic methods meets all needs for the identification of the SOZ since their time-resolution cannot capture the fast expansion of epileptic activity, making it impossible to differentiate between SOZ and networks of seizure propagation [6]. Therefore, research in past years has focussed on the analysis of EEG recordings with sufficient temporal resolution. In most of these studies, attention was paid to the analysis and localization of interictally recorded epileptiform discharges (IED) with or without simultaneous functional MRI [6-10]. However, since the source of interictal epileptiform activity does not necessarily coincide with the SOZ [11,12], methods analyzing ictal activity in the EEG would probably be more accurate in determining the location and extension of the SOZ as well as studying the subsequent epileptic networks. 

Different methods of EEG source analysis in time and frequency domain have been applied to characterize both the SOZ and epileptogenic network during focal epileptic seizures. The methods used range from ictal spatiotemporal dipole modeling (ISDM) [13], time frequency analysis [14], fast Fourier transformation (FFT), dipole approximation [15,16], to voltage-map dependent 3-dimensional source reconstruction [17]. Another novel solution to analyze the source of neuronal activity is dynamic imaging of coherent sources (DICS). DICS investigates neuronal interactions by imaging power and coherence estimates of oscillatory brain activity using a spatial filter [18,19]. The coherence can reveal the functional connectivity between brain regions involved in an epileptic seizure. Since the coherence analysis yields no information about the directionality of information flow, it can be combined with the renormalized partial directed coherence (RPDC), a method which describes the direction of information flow from one signal to another [20]. A combination of both methods would therefore allow the analysis of both the functional (correlation between regional activities) and effective (hierarchical relation and information flow between involved regions) connectivity of the different neuronal sources involved in epileptic seizures. As shown recently, DICS can be successfully applied to localize SOZ in patients with pharmacoresistant focal epilepsy based on high-frequency components of interictal epileptiform discharges [21]. It also can exhibit networks being activated during generalized seizures such as absences [22]. 

In the present study, DICS was used in combination with RPDC to analyze ictal EEG recordings in children with pharmacoresistant epilepsies who had undergone presurgical evaluation. The aim of the study was to address the following questions: Is it possible to determine the SOZ by using DICS? Is there a correlation between seizure semiology and the functional connectivity of neuronal structures during the seizure? And, are there significant differences in the effective connectivity of networks involved at the beginning and in the middle part of the seizure?

## Methods

### Subjects

Between November 2005 and June 2008, 26 children with pharmacoresistant, symptomatic, or cryptogenic focal epilepsy underwent our presurgical program in the Department of Neuropediatrics at the University Hospital of Schleswig-Holstein, Campus Kiel. From this group, six boys and five girls, aged from 1.1 - 18.67 years (mean age: 9.6±6.9 years) at the time of operation, were chosen for our study according to the following inclusion criteria: (1) clear epileptogenic focus in long-term video-EEG; (2) consistency of PET, ictal SPECT, and long-term video-EEG as determined by visual inspection; and (3) lack of large lesions which could substantially attenuate results of the source analysis (mostly cysts). Clinical characteristics of these patients are shown in Table 1. All diagnoses were made according to the guidelines of the International League Against Epilepsy (Commission on Classification and Terminology of the International League Against Epilepsy [ILAE], 1989; for revision, see 23). All included patients had only one type of seizures. The clinical outcome after surgery was assessed using the classification system proposed by [24]. The good postoperative outcome was defined according to the criteria of the Engel class I (free of disabling seizures) and Engel class IIa (immediately after surgery seizure free, than in follow-up almost seizure free). The neurological examination and structural MRI (high-resolution T1-, T2-, FLAIR-T2 and diffusion-weighted imaging) were performed before inclusion in the study. Results of the source analysis of interictal epileptiform discharges which was done in some patients (patients 1 - 6) were published previously [25]. The study was approved by the Ethics Committee of the Faculty of Medicine, University of Kiel, Germany. All participants and their parents were instructed about the study, and written informed consent according to the Declaration of Helsinki (current version, 1996) on biomedical research involving human subjects (Tokyo amendment) was obtained.

**Table 1:** Demographic details and clinical characteristics of the patients.

**Case Nr**	**Age**	**Sex**	**AED**	**structural MRI**	**Diagnosis**	**interictal EEG**	**Ictal EEG**
1	1.5	m	VPA	normal	cryptogenic TLE right	multifocal	temporal right
2	5.33	m	OXC	FCD right	symptomatic TLE right	temporal right	temporal right
3	17.17	f	LTG	normal	cryptogenic FLE left	frontal left	frontal left
4	3.83	m	VPA OXC	multiple cortical dysplasia	symptomatic FLE left	frontal left + right	frontal left
5	14.67	f	OXC TPM	dysplasia of right Uncus	symptomatic TLE right	fronto-temporal right	temporal right
6	17.5	f	CMZ LTG	tumor of amygdala and caput hippocampi left, mesial sklerosis of corpus hippocampi	symptomatic TLE left	temporal left	temporal left
7	15.08	f	VPA LTG	less volume in the left hemisphere; postcentral focal cortical dysplasia; subependymal heterotopy of the temporal horn left	symptomatic focal epilepsy by complex brain malformation left	parieto -temporo -occipital left	parieto-occ left
8	4.33	m	VPA LTG	dysplasia of gyrus parahippocampalis and temporal-occipitalis; dysplasia of temporal lobe left	symptomatic epilepsy temporal-occ. Left	multifocal; 50% temporal-occipital left; 40% parieto-occipital right	parieto-occ left
9	1.08	m	SM VBT	asymmetric myelinisation, left temporo-occipital more myelin; left FCD	symptomatic TLE left	temporo-occ.left	temporo-occ left
10	18.67	f	LEV VBT	tuberous sclerosis, genetic defect TSC-1 Gene;	symptomatic FLE left	temporal left	frontal left
11	6.42	m	GBP	FCD Type1B, hippocampus sclerosis	symptomatic TLE left	fronto -temporal left	temporal left

Abbreviation: AED = Antiepileptic Drug, VPA = Valproat, ESM = Ethosuximid, ZSM = Zonisamid, GBP = gabapentine, OXC = Oxcarbazepine, SM = Sultiam, LTG = Lamotrigine, TPM = Topiramat, CMZ = Carbamazepine, TLE = temporal lobe epilepsy, FLE = frontal lobe epilepsy, FCD = Focal Cortical Displasia, ILAE Ia = complete seizure freeness, IIa = initially seizure free, meanwhile occasional seizures, IIb = occasional seizures, Iva/b = no improvement; significant reduction of seizures (a)/ no significant reduction of seizures (b); follow-up time in months

### EEG recording

The EEG data were taken from the clinical long-term monitoring (LTM) which was carried out for at least 48 hours using a Nihon Kohden video-EEG system (Nihon Kohden, Japan, sampling rate 2000 Hz for each channel, filters 0.01 - 75 Hz, Ag/AgCl electrodes, electrode impedance < 5 kOhm). Depending on the patient, 38 to 50 electrodes were placed on the scalp according to the 10-10 international system. 

### EEG analysis and selection of EEG epochs

For each patient, the EEGs were evaluated by two experienced neurophysiologists independently, and a seizure typical for each patient was chosen. EEG segments for the beginning of the seizure and the middle part of the seizure were selected in correspondence with [26]. For the beginning of the seizure, EEG segments from 5 seconds before to 5 seconds after seizure onset were extracted. The seizure onset was defined as a first symptom detectable on video. In patients 1, 4 and 8 the seizure were very short (

< 3 sec). In these patients short segments with the 1 second’s duration were selected around the seizure onset. The frequency band at the beginning of the seizure was defined based on results of fast Fourier analysis [27]. The leading frequency with maximal global field power, which was consistent across three evaluated seizures, represented the frequencies of the seizure onset and was used for the further source analysis in the frequency domain [15]. In the same way, a data set of 10 seconds’ duration was chosen from the middle part of the seizure in all patients except of patients 1, 4 and 8. In these three patients, segments of 1 second duration were analysed. The middle part of the seizure was defined as a period around a middle time point between the first and the last symptom visible on video. The dominating frequency with the strongest power in the middle part of the seizure ranged individually (see table 2). This individual frequency was taken for the further source analysis. The termination of the seizure was not analysed because of the problematic and vague definition of the end of the seizure. There were no overlapping segments for seizure begin and middle part

 of the each particular seizure analysed (even in patients with short seizures).

**Table 2:** Operation procedure, frequency range analyzed for the beginning and middle of the seizures, DICS primary source, clinical outcome (- C - concordant; -DC - discordant).

**Case Nr**	**operation procedure**	**ILAE outcome (follow-up time)**	**Duration of analyzed seizure**	**Frequency analyzed at seizure begin**	**Frequency analyzed at seizure middle**	**DICS primary source**
1	hemisphaerotomy right	Ia (26)	1,8 sec	12-20Hz	2Hz	Temporal right - C -
2	2/3 resection temporal lobe right	IIb (12)	97sec	3-5Hz	2-4Hz	Temporal right - DC -
3	frontal lobe left	Ia (12)	30sec	16-20Hz	16-20Hz	Frontal left - C -
4	frontal lobe left	Ia (10)	1,6sec	16-20Hz	16-20Hz	Frontal left - C -
5	lesionectomy right temp	Ia (14)	32sec	2-3Hz	5-6Hz	Uncus right - C -
6	lesionectomy left temp mesial	Ia (24)	118sec	1-4Hz	1-4Hz	Temporal left - DC - Second source - C -
7	Multilobectoma TPO left (central and frontal regions were spared out)	IIa (6)	120sec	4-5Hz	4-5Hz	Occipital left - C -
8	Multilobecomy temporo-parieto-occipital left	Ia (6)	3sec	16-20Hz	2-3Hz	Parieto-occipital left - C -
9	multilobectomy left temporo-parieto-occipital	Ia (24)	30sec	4-7Hz	4-7Hz	Temporal left - C -
10	frontal lobe left	IVb (6)	51sec	1-4Hz	16-20Hz	Frontal left - DC -
11	2/3 resection of temporal lobe left	IVa (6)	40sec	2-3Hz	4-5Hz	Temporal left - DC -

### Source Analysis

DICS was used to find the sources of epileptic activity in the brain. The DICS analysis was performed in a blinded fashion so that the analyst did not know the patients’ diagnoses. DICS is efficient in localizing the coherent network of sources for a specific frequency band [18,19]. The spatial filtering used in this algorithm gives the ability to do further analysis on the time series extracted from these source regions. However, the temporal resolution of this method is significantly lower than of EEG analysis in time domain. Long data segments are needed to achieve an appropriate signal-to-noise ratio, especially for analysis of deep sources as done in this study. This is a clear disadvantage of DICS. 

In order to locate the origin of specific EEG activity seen on the scalp, two problems needed to be solved, namely the forward and inverse problem. The forward problem is the computation of the scalp potentials for a set of neural current sources. It is solved by estimating the lead-field matrix with specified models for the brain. In this study, the brain was modeled by a more complex, five-concentric-spheres model with a single sphere for each layer corresponding to the white matter, grey matter, cerebral spinal fluid (CSF), skull and skin. The standard conductivity values were used for all the five layers and they were 0.33 S/m for scalp, CSF and gray matter. The conductivity value for skull was 0.0042 S/m and 0.31 S/m for the white matter [28]. The models used for the forward computation are multilayer anisotropic spheres in which the innermost shell is considered to be anisotropic. The volume conductor model was created using standard T1 magnetic resonance images. The template model created was then warped onto the standard head model. The open-source software "FieldTrip" was used [29]. The head was modeled by entering the radius and the position of the sphere with the standard electrode locations. In order to map the current dipoles in the human brain to the voltages on the scalp, the lead-field matrix (LFM) needs to be calculated. The LFM contains information about the geometry and the conductivity of the model. The inverse problem is the quantitative estimation of the properties of the underlying neural current sources of EEG activity. The neural activity is modeled as a current dipole or sum of current dipoles. The power and coherence at any given location in the brain can be computed using a linear transformation which, in our case, is the spatial filter. In this study, the linear constrained minimum variance (LCMV) spatial filter was used which relates the underlying neural activity to the electromagnetic field on the surface. The main aim of the LCMV method was to design a bank of spatial filters that attenuates signals from other locations and only permits signals generated from a particular location in the brain. The DICS method employed a spatial filter algorithm to identify the spatial power maximum or coherence in the brain for a particular frequency band. The spatial extent of each voxel is defined to be 5mm in this study. In this study, we use a regularization value of α = 0.001. This value is chosen due to the reason that it is tested in simulations and successfully applied in real data not yielding spurious spatial extension of sources for a voxel definition of 5 mm [30,31]. The brain region representing the strongest power in a specific frequency band can subsequently be used as a reference region for cortico-cortical coherence analysis. In order to create topographic maps, the spatial filter is applied to a large number of voxels covering the entire brain using a voxel size of 5 mm. The individual maps of coherence were spatially normalized and interpolated on a standard T1 brain in SPM8. 

For each patient, the brain source with the strongest power in the individual band (see "Selection of EEG epochs") was identified and defined as the reference region for further coherence analysis between brain areas. Since the coherence of a reference region with itself is always 1, the reference region was projected out of the coherence matrix, and further coherent areas were found. The statistical significance of the identified coherent sources was tested by a within-subject surrogate analysis. The surrogate analysis was done by shuffling one second time windows of the EEG time series to create the null data. But here the permutation is done 100 times using the Monte Carlo test of permutation from the Fieldtrip toolbox [29]. The p-value is estimated for each of these 100 permutations and the 99 percentile value is then set as the threshold for the original time series for each individual subject separately. Once coherent brain areas were identified, their activity was extracted from the surface EEG by the spatial filter [32].

### Directionality analysis

Coherence analysis only reveals components that are common to two signals in the frequency domain. It does not give the direction of information flow between the two signals. In this study we applied renormalized partial directed coherence (RPDC), a technique used in the frequency domain to detect the direction of information flow from one signal to the other and vice versa. The RPDC method applies a multivariate (MVAR) autoregressive modeling approach which is based strictly on causality (i.e., not taking into account zero-lagged or instantaneous influences). The method was used to model the pooled source signal estimates by an autoregressive process to obtain the coefficients of the signals in the defined frequency band. The open source Matlab (The MathWorks Inc., Natick, MA, USA) package ARFIT [33,34] was used to estimate the autoregressive coefficients from the spatially filtered source signals. In order to obtain these coefficients, the correct model order needs to be chosen which is estimated by minimizing the Akaike information criterion (AIC) and gives the optimal order for the corresponding signal [35]. The AIC is a measure of the relative goodness of fit which has the minimum loss of information of a resulting statistical model with an optimal order for the corresponding model[35]. After estimating the RPDC values, the significance level is calculated from the applied data using a bootstrapping method [36]. In the bootstrapping method we shuffle one second time window segments of the original pooled source signal time series 19 times and the mean RPDC value is the null data. The 20^th^ time we estimate the RPDC with the pooled original source signal time series and test against the surrogate mean value to indicate whether it is a significant value.

Patients 1, 4 and 8 were excluded from the RPDC analysis because of a very short duration of seizure which may bias the results.

### Assessment of concordance

To assess the concordance of the primary source as found by DICS, we chose the following definition (see also 37,38): If the primary source corresponded with the resected area on the sub-lobal level as revealed by the postoperative MRI, the results of DICS analysis were considered to be concordant (C). If not or incomplete correspondence between the primary source and resected area was found (for example, of the source extended over the border of the resected area), the results were characterized as discordant (DC). 

## Results

### Patients

An overview of the clinical data of the eleven patients included in this study is given in Table 1. Nine of 11 patients suffered from symptomatic focal epilepsies, 6 of which were temporal lobe epilepsies (TLE), 2 of which were frontal lobe epilepsies (FLE) and one presented with multifocal epilepsy with the left parieto-occipital seizure onset zone. In the remaining 2 patients, cryptogenic focal epilepsy was diagnosed (one TLE and one FLE). After operation, 7 of 11 patients became seizure-free (Engel Ia). Of the remaining 4 patients, one had a postoperative outcome Engel IIa (initially seizure-free, but later with occasional seizures; Patient 7), IIb (occasional seizures; Patient 2), IVa (significant seizure reduction; Patient 11) and IVb (no significant reduction of seizures; Patient 10), respectively. 

### DICS of the seizure onset

Analyzing the point of the seizure onset, the localization of the source with the strongest power, was in accordance with the electro-clinical localization of the point of seizure onset, as well as the operation field as seen on the postoperative MRI in 7 of 11 (64%) patients studied. All of these patients were seizure-free after surgery. In one patient, the second strongest source was concordant to the electro-clinical localization (Patient 6; see Table 2). One patient was seizure free immediately after the operation, but developed seldom but well drug controlled seizures later (Patient 7). In patients 2, 10 and 11 the primary source was found much closed to the resected area. However, in all these cases the primary source was larger than the resected brain region, and the correspondence was incomplete. Therefore, in these patients the results were rated as discordant. And all of these three patients did not achieve postoperative seizure freedom. 

The first two sources as found by DICS were predominantly in cortical structures (including hippocampus 3 patients; 27%); subsequent sources also included subcortical structures, such as the thalamus (6 patients; 55%), caudate nucleaus and Nucl. subthalamicus (4 patients; 36%) as well as cerebellum (5 patients; 45%). A detailed overview of the neural structures involved in the beginning of the seizure as revealed by DICS is shown in Table 1 of the supplementary material. Considering the different seizure semiologies: four patients lost consciousness during seizure (Patients 3, 6, 10, and 11); five patients displayed motoric automatisms (Patients 5, 6, 7, 10, and 11); and three patients had infantile spasms (Patients 1, 4, and 8). All four patients with loss of consciousness had a source in the thalamus/Nucl. subthalamicus as one of the sources found by DICS. The three patients without loss of consciousness (and without infantile spasms) had the thalamus as one of the subsequent sources. It was impossible to make any conclusion concerning clinical value of other subcortical sources (cerebellum or brainstem). 

### DICS of the middle part of the seizure

Analysis of the middle part of the seizure yielded similar sources as those obtained at the beginning of the seizure. The localization of the source with the strongest power was in concordance with the electro-clinical localization of the point of seizure onset as well as the operation field as seen on the postoperative MRI in the same 7 of 11 (64%) patients studied. In Patient 6, the second strongest source was concordant to the electro-clinical localization. In contrast to the sources of the seizure onset, fewer subcortical sources were found. The thalamus and nucl. subthalamicus were found to be sources in 8 patients (72%); the cerebellum and uncus in 1 patient (9%). No novel subcortical sources were found (see Table 1, suppl. material). The four patients that lost consciousness during the seizure still had a thalamic source. In the other four patients without loss of consciousness (and without infantile spasms) the thalamus was found to be one of the subsequent sources. There was no correspondence between other subcortical sources and any seizure semiology.

### Directionality of information flow in seizure networks

**Figure 1:**
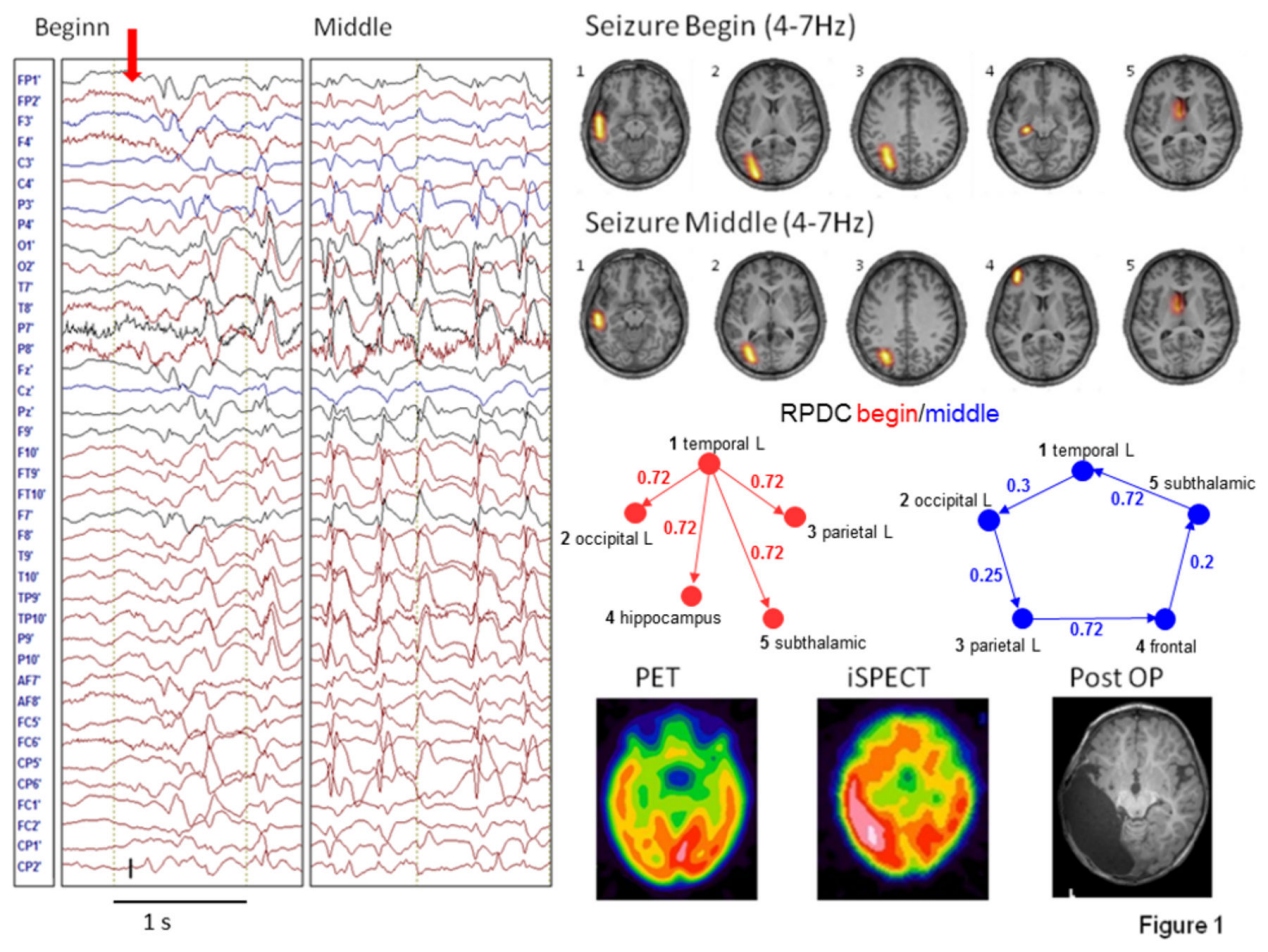
The time series of a short seizure (left) in Patient 9, DICS, and RPDC results of the beginning and middle of the seizure (dashed line in the bar plot of the RPDC indicates the significance level), respectively (top three lines). PET, SPECT, and postoperative MRI results (bottom line, from left to right).

As for the onset of the seizures, the results of the RPDC showed a possible pathway of seizure propagation, since the significant direction of information flow was always from the primary source to the other coherent sources (Figure 1 and Figure S 1-11). The results of the RPDC of the middle part of the seizure showed possible mechanisms of seizure perpetuation: the information flows in circles between all involved sources (Figure 1 and Figure S 1-11). 

### Example of a patient

Figure 1 shows the results of the patient 9. These includes a segment of the EEG recording taken for analysis, the primary and coherent sources as found for the seizure begin and middle part of the seizure, the directionality of information flow, the postoperative MRI PET and ictal SPECT results. Patient 9 (Figure 1) had a cryptogenic TLE left with 90% of the epileptic discharges in the LT-EEG being temporo-occipital left (T3/O1). The clinical picture of the seizures comprised pausing of all movement, wide-eyed-ness, tonic stretching of the right arm, cloni of the right hand. Postictally, the patient displayed paresis of the right arm, ptosis, and convergent strabismus on the right side. After the seizures (mostly only 10-15sec in duration), tonic spasms of the right side and nystagmus could be observed for up to 9 minutes. In the MRI there was an asymmetrical myelination pattern with accelerated myelination in the left temporo-occipital region. The ictal SPECT showed circumscribed enhancement in the left temporal (occipital) region, the interictal PET demonstrated a moderately increased enhancement in the left temporal region. Two years after the left temporo-parieto-occipital multilobectomy the patient was still seizure-free. The sources determined by DICS for the beginning of the seizure include the main electroclinical localization of the epileptogenic focus (temporal and occipital left), parietal left, the hippocampus and Nucl. Subthalamicus. In the middle part of the seizure, no source in the hippocampus was found, but an additional source in the left frontal region was found instead. The results of the RPDC show the significant direction of information flow going from the primary (temporal left) source to all the subsequent sources at seizure onset and a circular flow of information from the primary source via all subsequent sources back to the primary source again for the middle of the seizure.

## Discussion

In the present study, two main hypotheses were studied: (1) DICS is a powerful technique to characterize seizure onset zones in pediatric partial epilepsies; (2) the functional and effective connectivity between brain regions involved into the epileptic network are different by the generation and during the perpetuation of epileptic seizures, indicating different mechanisms in these phases of ictogenesis. Both hypotheses were confirmed. Firstly, in patients who became seizure-free after epileptic surgery, the study demonstrated good correspondence between results of the DICS-based source analysis, electro-clinical localization of seizures, and the resected brain area. In patients who showed discordant results, a poor postoperative outcome was observed. And secondly, although similar sources were described for the beginning and middle part of the seizure, the significant information flow at seizure onset occurred from the first dominating source (in all cases fitting the SOZ) to all other sources demonstrating propagation. In the middle part of the seizures the information flow went in circles between all involved sources, indicating possible pathways of seizure perpetuation. Therefore, as hypothesized, different patterns of effective connectivity were observed during the phase of generation and the phase of perpetuation of epileptic seizures.

### Seizure onset zone

As discussed earlier, different methods have been developed to improve localization of the seizure onset zone based on ictal surface EEG recordings. Studies have shown that these methods are successful in localization of the SOZ and can even achieve a relatively high sensitivity, given that the results of these methods were concordant with the electro-clinical localization of the SOZ in 78 - 100% of cases. This high sensitivity corresponds with the results of our study. The strongest sources corresponding to the operation field as seen on the postoperative MRI were found in almost all patients who became seizure-free after epileptic surgery (in one patient the second source corresponded with the resection area). This means that, in all patients in whom the SOZ was resected successfully, DICS was able to find a source corresponding with the resected brain region. All three patients, in whom the primary and secondary sources were incompletely concordant with the postoperative MRI, the postoperative outcome was poor. It can be suggested that the surgical intervention did not remove the whole SOZ, probably shown by the primary source of the DICS. Because the postoperative MRI and clinical outcome after epilepsy surgery represent the modern gold standard for validation of localization techniques for the SOZ [1,38], this study indicates that DICS can be a valid method and used successfully to characterize SOZ in children with partial seizures.

As suggested before, partial seizures start in the SOZ and then propagate to other brain regions involving a complex neuronal epileptic network into the hyper-synchronous activity [4]. In this study, subsequent coherent sources as determined by DICS included other cortical areas in the same hemisphere as the primary source and subcortical structures such as the thalamus, Nucl. Subthalamicus and cerebellum. The thalamus plays an important role in the genesis and spreading of temporal lobe seizures [39-41]. In our study, in 5 out of 7 patients with TLE and in 1 out of 4 patients with extra temporal epilepsies the thalamus was involved into the coherent network responsible for the onset of the seizure. In patients with TLE, thalamo-cortical synchronization was discussed as an important mechanism underlying the reduction of consciousness often accompanying complex partial seizures [26,42]. In our study, 4 of 7 patients with TLE displayed a loss of consciousness and in only 2 of these patients was the thalamus involved in the coherent network. Therefore, no clear association between sources in the thalamus and specific seizure semiology, such as reduction of consciousness during complex partial seizures, was found in the present study. 

The same is true for other deep sources. In five patients, the cerebellum was described as a coherent source. Cerebellar ictal hyperperfusion has been observed in patients with partial and generalized seizures [42]. The role of the cerebellum during seizures or interictal epileptiform discharges has been reviewed by [43], but the authors do not offer a specific hypothesis regarding its pathophysiological significance. The hippocampus and the uncus were described as a significant epileptogenic structure, especially in the context of TLE [2,3]. The role of the subthalamic nucleus in the epileptogenesis remains unclear, although it has been discussed as being a part of the epileptic network [44] and is repeatedly used as a target structure in deep brain stimulation studies for the treatment of epileptic encephalopathies [45]. In the present study, however, it was not possible to find convincing evidence that source in the cerebellum, Nucl. subthalamicus or uncus are associated with any specific clinical semiology or propagation pattern of the partial seizures observed in our patients. Therefore, our study is unable to contribute to our understanding of pathophysiological significance of the structures mentioned. 

### Epileptic network of seizure generation and perpetuation

Different studies have repeatedly shown that interictal epileptiform discharges and epileptic seizures are associated with hyper-synchronous activity in a complex epileptic network [4]. This network may consist of structures which are involved in the (1) generation and (2) propagation of epileptic activity (e.g., hippocampus and entorhinal cortex; see 2,4), (3) modulation of cortical excitability which predisposes neuronal assembles to hyper-synchrony (e.g., thalamocortical network; see 46), and (4) reactive functional changes in neuronal structures underlying pathological synchronization (e.g., interrupted activity in the default mode network; see 9,47). Different techniques such as fMRI, PET, and intracranial recording were able to describe complex epileptic networks during seizures [4,10,48-52] but were unable to show the influence of structures on each other within the network. By applying the RPDC to the sources determined by DICS, we demonstrated the direction of information flow between the different sources. Based on Granger causality, RPDC investigates the temporal dynamics of two time series and shows the dependence of one time series on another [20]. In doing so, we wanted to investigate the possible causal relationships between different structures involved in seizure generation. At seizure onset the significant direction of information flow always went from the primary source to the subsequent coherent sources. It is likely that the activity arising in the first source propagates to other sources. The RPDC analysis provided additional evidence that epileptic activity develops in focal epilepsies as a result of pathological synchronization in the circumscribed SOZ and then spreads to other brain regions involving cortical and subcortical structures in the epileptic network. In the patients investigated, the thalamus and other structures that exert a modulating influence on epileptic activity are involved secondarily to the activity in the SOZ. It can be suggested that such a complex neuronal network associated with seizure onset may represent individual differences in seizure semiology. We cannot answer this question positively, but further studies with more patients are needed to show possible correlations between individual epileptic networks and clinical features of epileptic seizures.

Surprisingly, the same structures involved in the generation of epileptic seizures can be found in the middle part of the seizures as well. The only difference between the beginning and middle part of the seizure is the significant information flow between sources. During the seizure, the activity is distributed circularly between sources. It seems likely that the circular information flow in the middle of the seizure may demonstrate a way of seizure perpetuation. Although the interpretation of results of the RPDC analysis is rather hypothetical, the results of RPDC analysis are exciting and innovative as they provide a new possibility to explain behavioral phenomena (for example during seizures). Differences between symptoms may be related not only to different brain structures responsible for any behavioral phenomenon, but also to different hierarchic relations between the same brain structures. This finding provides a new idea how to explain different clinical expressions in the course of a seizure shifting the main focus to the hierarchic organization of neuronal networks. Although exciting, this idea needs further confirmation.

### Limitations

Although we could show good localization power of DICS, this study also has some limitations. Firstly, the study revealed brain sources in deep brain structures such as the thalamus, Nucl. Subthalamicus, and hippocampus. It can be debated whether it is possible to identify sources deep in the brain based on recordings from the scalp. In previous MEG studies, subcortical sources have been detected by applying DICS to oscillatory signals (e.g. tremor) [18,19,53-55] and recently in EEG studies [56,57]. Moreover, DICS revealed sources in deep brain structures (thalamus) for oscillatory epileptic activity based on EEGs obtained simultaneously with functional MRI in patients with absence seizures and photo paroxysmal responses. The sources found in the thalamus corresponded with positive BOLD signal changes in the thalamus in all patients. Also other recent studies used fMRI results obtained in the same patients in order to validate results of DICS analysis and demonstrated a good correspondence between sources in deep brain structures and BOLD signal changes in the same brain areas [31,58]. Compared with other methods of source reconstruction, DICS has some advantages that increase its power in the detection of deep brain sources. DICS analysis does not try to explain the signal recorded on the scalp EEG by single sources but looks for sources that are coherent to a given reference point. Since only a specific frequency range of interest is analyzed, even small oscillatory activity in this frequency range can be detected. Additional aspects support the fact that DICS is also sensitive to deep sources. For example, simulation studies showed that a simulated source with a physiological signal-to-noise ratio placed in the diencephalon could be located correctly by DICS [59,60]. Moreover, different control calculations demonstrated a high reliability of DICS for the detection of deep sources: 1) Analysis of randomly chosen EEG segments of no interest (baseline) did not reveal the same coherent network as for EEGs segments with seizures; 2) Shifting the first source within the coherent network, for example to the thalamus, revealed the same coherent sources, whereas shifting of the first source to any other brain area destroyed the coherent network of sources (for a detailed description see 60. Additionally, it is worth drawing attention to the following point [60]: detected subcortical sources using a 30-channel EEG recording. Since the accuracy of source localization increases with the number of electrodes used [61], the detection power must be better in our study, because we used up to 50 electrodes. Nonetheless, other validation studies are needed; especially those which imply simultaneous recordings from deep brain structures and the scalp of patients, even if these studies are difficult to perform. Moreover, if the seizure onset zone is in deep brain structures the interpretation of results should be very careful when applying methods of source analysis. We still cannot exclude the possibility that DICS did not detect some deep sources, so we have always to consider additional possible undetected nodes in the epileptic network.

Secondly, the segments for the source analysis were chosen based on visual inspection of the seizure onset while segments from the middle part of the seizure were selected based on time. Because there is sometimes a discrepancy between the seizure onset on the video and seizure-related EEG changes (in some cases, EEG changes preceded clinical symptoms on the video), we have chosen the standardized procedure in the majority of patients taking 5 sec before and 5 sec after the seizure onset. This is a suboptimal procedure, since before the beginning of the seizure both normal oscillations and seizure activity were involved into the analysis. So, a bias due to the voluntary selection of segments cannot be excluded. However, even using this procedure, we were able to achieve a good correspondence between DICS and post-operative resection area. Moreover, the analysis of the middle part of the seizure which definitely included only seizure activity, revealed the same sources as the analysis of the beginning of the seizure in all patients. In previous studies, different methods for selection of appropriate segments for source analysis have been demonstrated [17,62,63]. Especially if the beginning of seizures may vary from seizure to seizure, a retest reliability of results has to be proven in the future [64].

Thirdly, using DICS it is very difficult to assess the expanse of brain area involved into the epileptic activity. Moreover, the seizure onset zone may be more complex than just one epileptogenic area in the brain and may include primary and secondary foci [65]. These limitations may explain why the patient 7 developed seizures in the course of her epilepsy despite of seizure freedom immediately after surgery and concordance between DICS results and the resected brain area. On the one hand, the SOZ was possibly larger that the resected area and the extent of the corresponding electrical source. On the other hand, secondary epileptogenesis and mirror foci may explain the occurrence of seizures after a successful surgery. In such a way, the validity of DICS can be compromised by the long-term outcome but remains acceptable if the short-term outcome will be taken into account. Note that even in patients 2, 10 and 11, who did not become seizure free after the surgery, there was a partial correspondence between the first source and the resected area. However, the first source was larger than the resected area. Therefore, the results were rated as discordant. It could be suggested that the SOZ was better represented by the DICS method and was larger than the resected brain area. However, this suggestion is too hazardous, and further studies are needed in order validate the value of DICS in assessing the expanse of brain area involved into the epileptic activity.

And finally, another weakness of our study is the inhomogeneity of patients. Although most patients investigated suffered from TLE, some patients with extra temporal epilepsies were included. Moreover, patients also differed in the clinical semiology of their epileptic seizures: some patients experienced a reduction of consciousness during seizures, others had only short spasms, while even others displayed motoric automatisms. However, because of this inhomogeneity, it was not possible to show a correlation between specific sources within the coherent network (for example, the source in the thalamus) and clinical expression as well as clinical propagation pattern of seizures (for example, loss of consciousness). In the future, more homogeneous groups of patients have to be investigated.

Despite these limitations, this study described for the first time functional and effective connectivity in the generation and perpetuation of different partial epileptic seizures in children. 

## Conclusion

Taken together, dynamic imaging of coherent sources (DICS) is a powerful tool for localizing the seizure onset zone as well as the subsequent cortical and subcortical structures involved in seizures using ictal EEG recordings. In combination with the renormalized partial directed coherence (RPDC), it is also possible to study the neuronal networks and causal relationships between sources at the beginning (phase of generation) as well as in the middle part (phase of maintenance) of the seizure. Before this method can be incorporated into the preoperative evaluation of patients with refractory epilepsies, future research remains to be done. For example, sources need to be located using realistic head models making the inclusion of patients with brain lesions into studies possible. Furthermore, more homogenous groups should be included to study possible correlations between seizure morphology and neuronal networks. 

## Supporting Information

Figure S1 **The networks of sources for the seizure begin in the (first row) followed in the (second row) for the seizure middle.** The postoperative MRI result is shown for patient 1.
(TIF)

Figure S2 **The networks of sources for the seizure begin in the (*first row*) followed in the (*second row*) for the seizure middle.** The (*third row*) with the (*bar plot in red*) shows the RPDC values for the seizure begin and the (*blue bar plot*) shows the RPDC values for the seizure middle. The postoperative MRI result is shown for patient 2. In this patient, the slice A. corresponds with the primary source. However, the resected area is shown on the lower slice B. which corresponds incompletely with the primary source. Therefore, the results were rated as discordant. The MRI slices showing the primary DICS source and the post-operative outcome do not coincide perfectly for following reasons: the DICS sources are shown on a standardized adult MNI brain. The post-OP MRI’s are made of children, some as small as 1,5 years of age, not lying perfectly straight in the scanner and with movement artifacts. Additionally the post-operative MRT’s were done with reduced number of slices due to which the selection of the same slice was impossible.
(TIF)

Figure S3 **The networks of sources for the seizure begin in the (*first row*) followed in the (*second row*) for the seizure middle.** The (*third row*) with the (*bar plot in red*) shows the RPDC values for the seizure begin and the (*blue bar plot*) shows the RPDC values for the seizure middle. The postoperative MRI result is shown for patient 3.
(TIF)

Figure S4 **The networks of sources for the seizure begin in the (*first row*) followed in the (*second row*) for the seizure middle.** The postoperative MRI result is shown for patient 4.
(TIF)

Figure S5 **The networks of sources for the seizure begin in the (*first row*) followed in the (*second row*) for the seizure middle.** The (*third row*) with the (*bar plot in red*) shows the RPDC values for the seizure begin and the (*blue bar plot*) shows the RPDC values for the seizure middle. The postoperative MRI result is shown for patient 5.
(TIF)

Figure S6 **The networks of sources for the seizure begin in the (*first row*) followed in the (*second row*) for the seizure middle.** The (*third row*) with the (*bar plot in red*) shows the RPDC values for the seizure begin and the (*blue bar plot*) shows the RPDC values for the seizure middle. The postoperative MRI result is shown for patient 6.
(TIF)

Figure S7 **The networks of sources for the seizure begin in the (*first row*) followed in the (*second row*) for the seizure middle.** The (*third row*) with the (*bar plot in red*) shows the RPDC values for the seizure begin and the (*blue bar plot*) shows the RPDC values for the seizure middle. The postoperative MRI result is shown for patient 7.
(TIF)

Figure S8 **The networks of sources for the seizure begin in the (*first row*) followed in the (*second row*) for the seizure middle.** The postoperative MRI result is shown for patient 8.
(TIF)

Figure S9 **The networks of sources for the seizure begin in the (*first row*) followed in the (*second row*) for the seizure middle.** The (*third row*) with the (*bar plot in red*) shows the RPDC values for the seizure begin and the (*blue bar plot*) shows the RPDC values for the seizure middle. The postoperative MRI result is shown for patient 9.
(TIF)

Figure S10 **The networks of sources for the seizure begin in the (*first row*) followed in the (*second row*) for the seizure middle.** The (*third row*) with the (*bar plot in red*) shows the RPDC values for the seizure begin and the (*blue bar plot*) shows the RPDC values for the seizure middle. The postoperative MRI result is shown for patient 10.
(TIF)

Figure S11 **The networks of sources for the seizure begin in the (*first row*) followed in the (*second row*) for the seizure middle.** The (*third row*) with the (*bar plot in red*) shows the RPDC values for the seizure begin and the (*blue bar plot*) shows the RPDC values for the seizure middle. The postoperative MRI result is shown for patient 11.
(TIF)
