# Supplementary figures and images for: Dynamic Imaging of Coherent Sources Reveals Different Network Connectivity Underlying the Generation and Perpetuation of Epileptic Seizures
Source: PLoS One. 2013 Oct 23;8(10):e78422. doi: 10.1371/journal.pone.0078422 (PMC3806832; doi:10.1371/journal.pone.0078422)

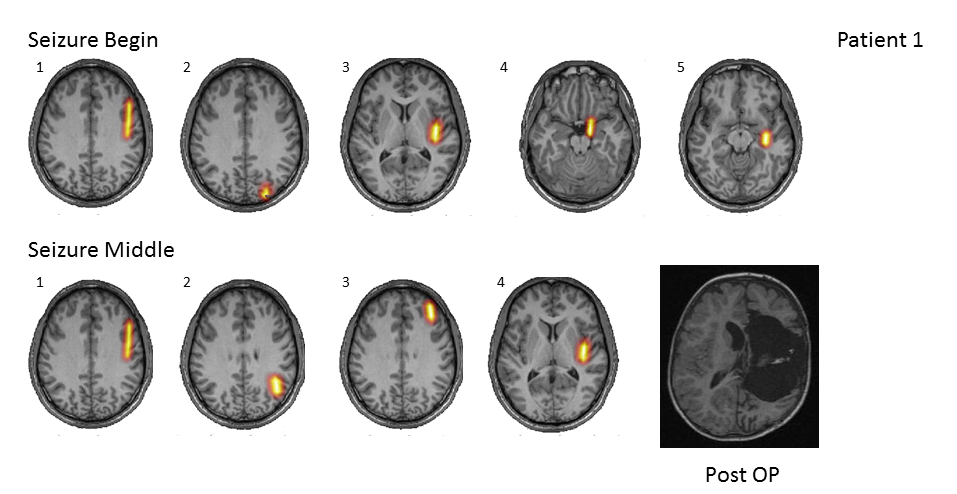

Supplement: Figure S1 — The networks of sources for the seizure begin in the (first row) followed in the (second row) for the seizure middle. The postoperative MRI result is shown for patient 1. (TIF) [file pone.0078422.s001.tif]

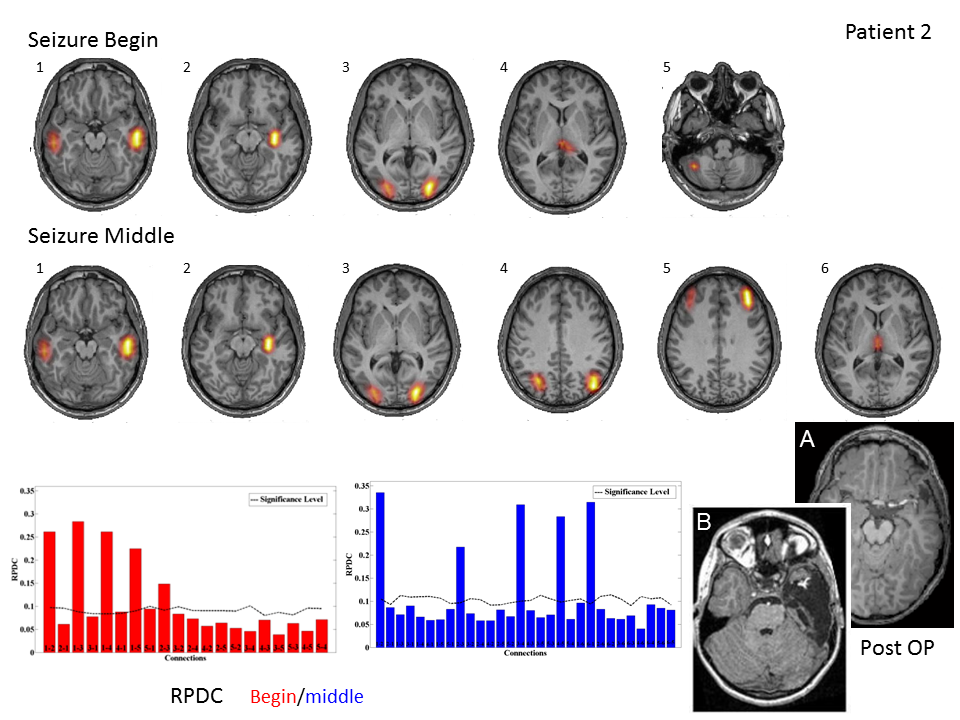

Supplement: Figure S2 — The networks of sources for the seizure begin in the (first row) followed in the (second row) for the seizure middle. The (third row) with the (bar plot in red) shows the RPDC values for the seizure begin and the (blue bar plot) shows the RPDC values for the seizure middle. The postoperative MRI result is shown for patient 2. In this patient, the slice A. corresponds with the primary source. However, the resected area is shown on the lower slice B. which corresponds incompletely with the primary source. Therefore, the results were rated as discordant. The MRI slices showing the primary DICS source and the post-operative outcome do not coincide perfectly for following reasons: the DICS sources are shown on a standardized adult MNI brain. The post-OP MRI’s are made of children, some as small as 1,5 years of age, not lying perfectly straight in the scanner and with movement artifacts. Additionally the post-operative MRT’s were done with reduced number of slices due to which the selection of the same slice was impossible. (TIF) [file pone.0078422.s002.tif]

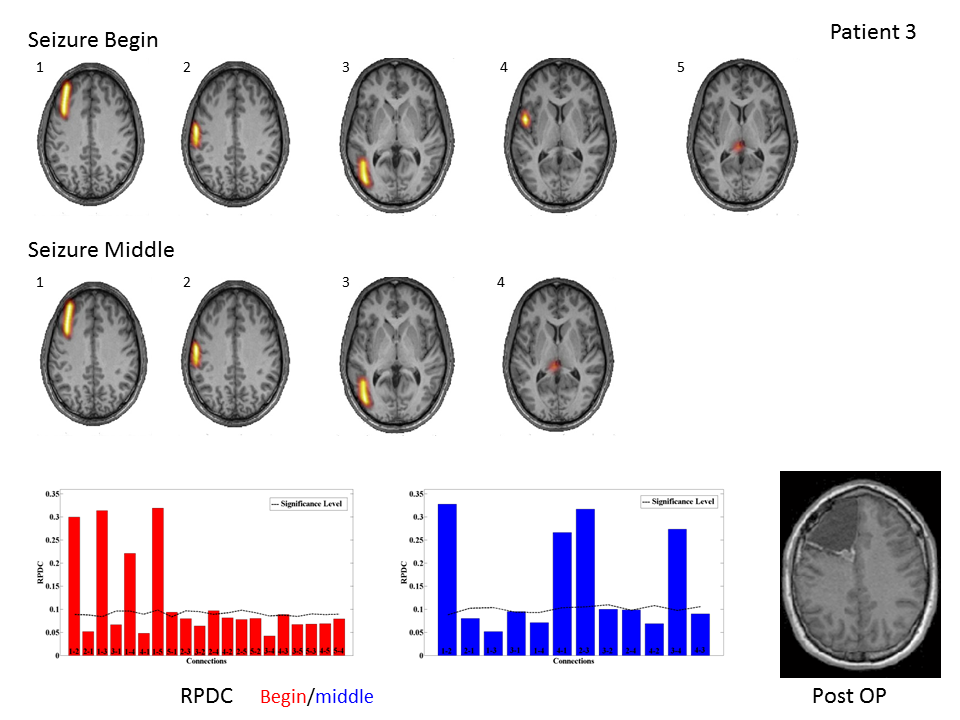

Supplement: Figure S3 — The networks of sources for the seizure begin in the (first row) followed in the (second row) for the seizure middle. The (third row) with the (bar plot in red) shows the RPDC values for the seizure begin and the (blue bar plot) shows the RPDC values for the seizure middle. The postoperative MRI result is shown for patient 3. (TIF) [file pone.0078422.s003.tif]

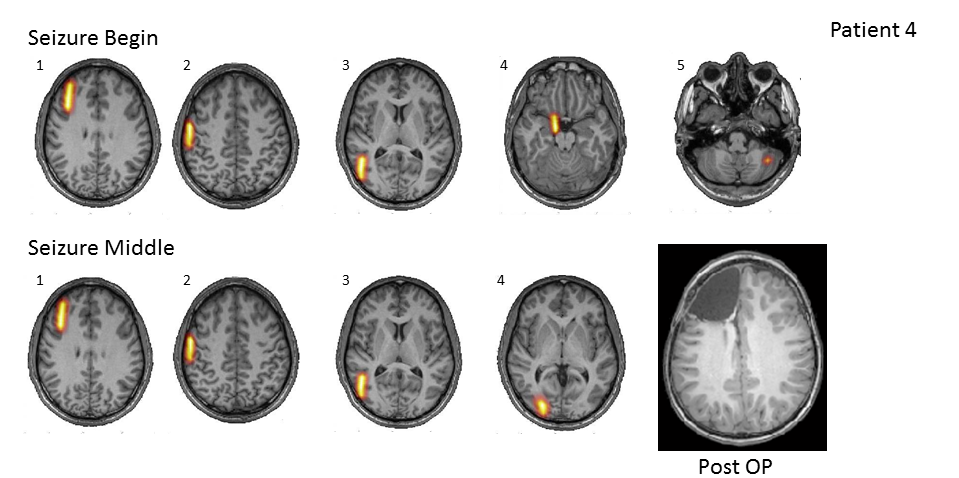

Supplement: Figure S4 — The networks of sources for the seizure begin in the (first row) followed in the (second row) for the seizure middle. The postoperative MRI result is shown for patient 4. (TIF) [file pone.0078422.s004.tif]

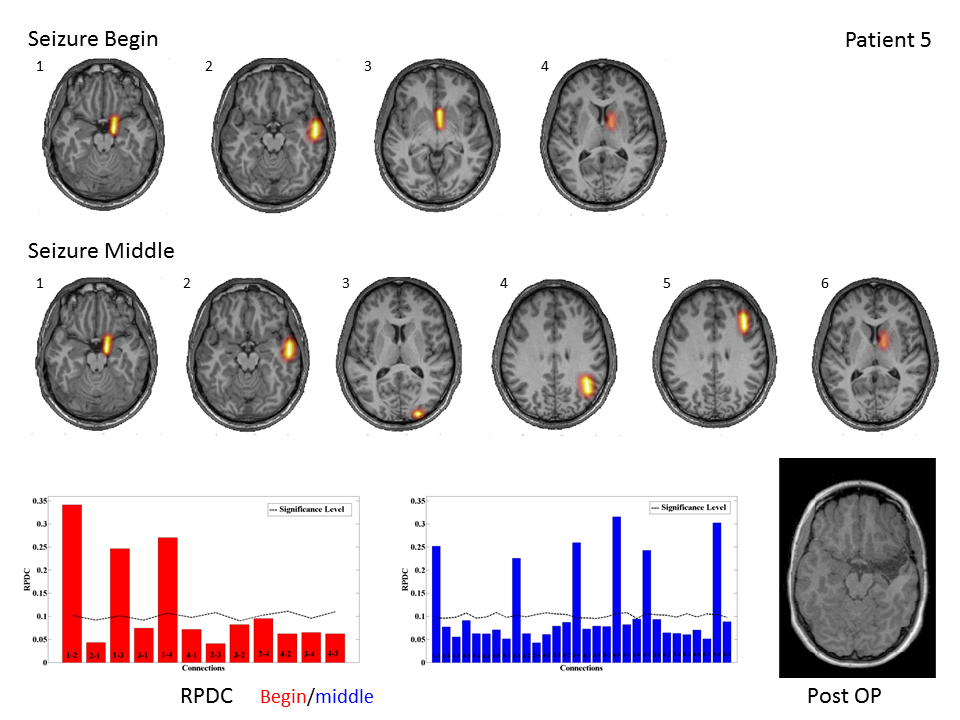

Supplement: Figure S5 — The networks of sources for the seizure begin in the (first row) followed in the (second row) for the seizure middle. The (third row) with the (bar plot in red) shows the RPDC values for the seizure begin and the (blue bar plot) shows the RPDC values for the seizure middle. The postoperative MRI result is shown for patient 5. (TIF) [file pone.0078422.s005.tif]

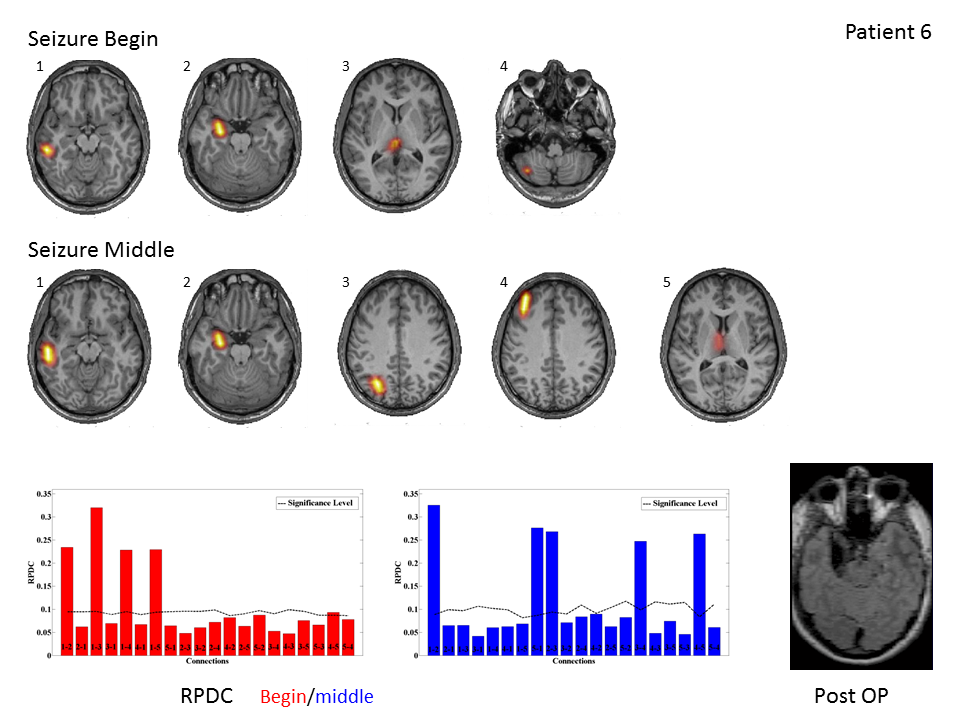

Supplement: Figure S6 — The networks of sources for the seizure begin in the (first row) followed in the (second row) for the seizure middle. The (third row) with the (bar plot in red) shows the RPDC values for the seizure begin and the (blue bar plot) shows the RPDC values for the seizure middle. The postoperative MRI result is shown for patient 6. (TIF) [file pone.0078422.s006.tif]

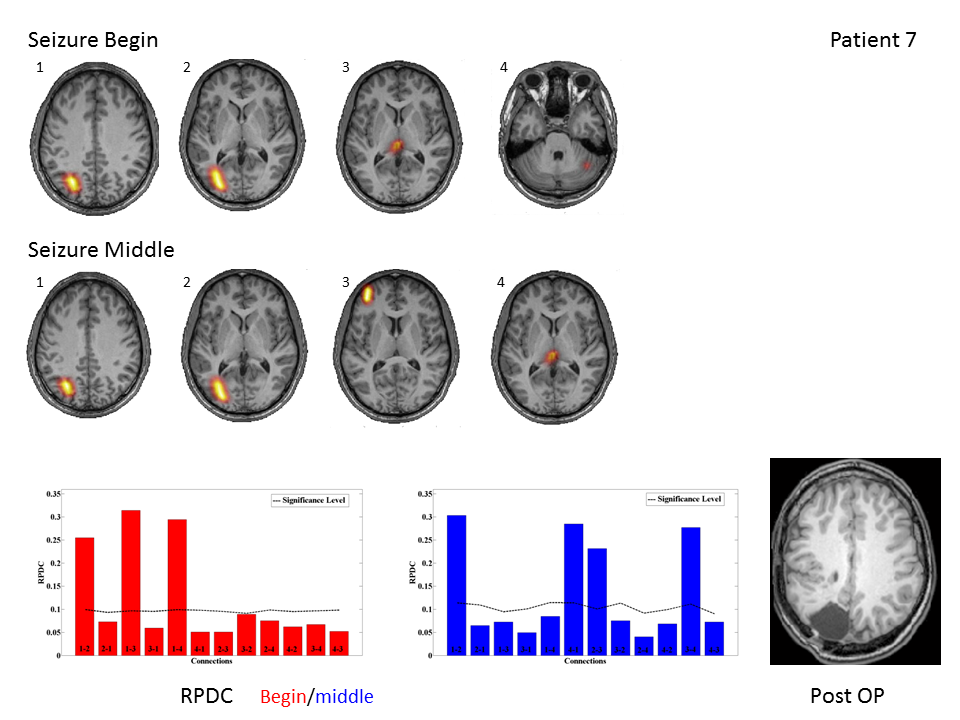

Supplement: Figure S7 — The networks of sources for the seizure begin in the (first row) followed in the (second row) for the seizure middle. The (third row) with the (bar plot in red) shows the RPDC values for the seizure begin and the (blue bar plot) shows the RPDC values for the seizure middle. The postoperative MRI result is shown for patient 7. (TIF) [file pone.0078422.s007.tif]

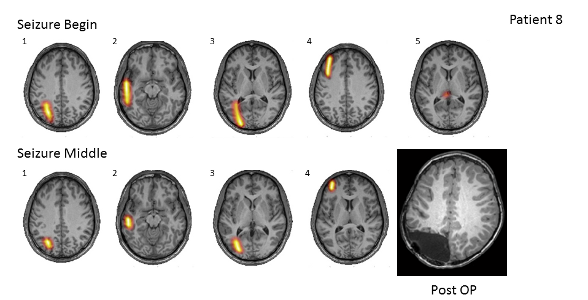

Supplement: Figure S8 — The networks of sources for the seizure begin in the (first row) followed in the (second row) for the seizure middle. The postoperative MRI result is shown for patient 8. (TIF) [file pone.0078422.s008.tif]

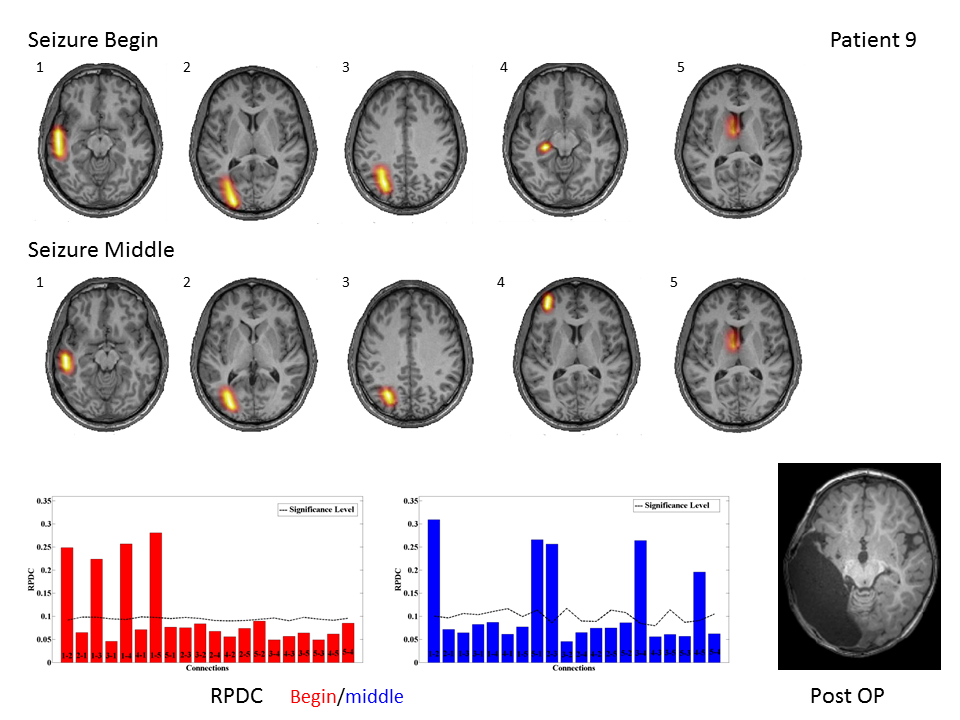

Supplement: Figure S9 — The networks of sources for the seizure begin in the (first row) followed in the (second row) for the seizure middle. The (third row) with the (bar plot in red) shows the RPDC values for the seizure begin and the (blue bar plot) shows the RPDC values for the seizure middle. The postoperative MRI result is shown for patient 9. (TIF) [file pone.0078422.s009.tif]

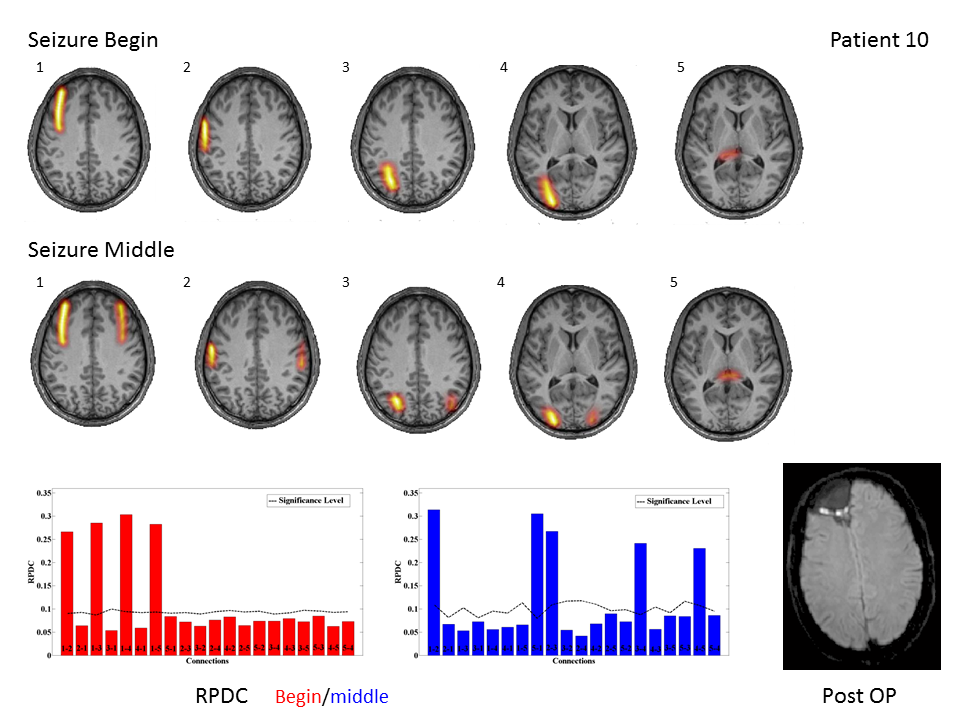

Supplement: Figure S10 — The networks of sources for the seizure begin in the (first row) followed in the (second row) for the seizure middle. The (third row) with the (bar plot in red) shows the RPDC values for the seizure begin and the (blue bar plot) shows the RPDC values for the seizure middle. The postoperative MRI result is shown for patient 10. (TIF) [file pone.0078422.s010.tif]

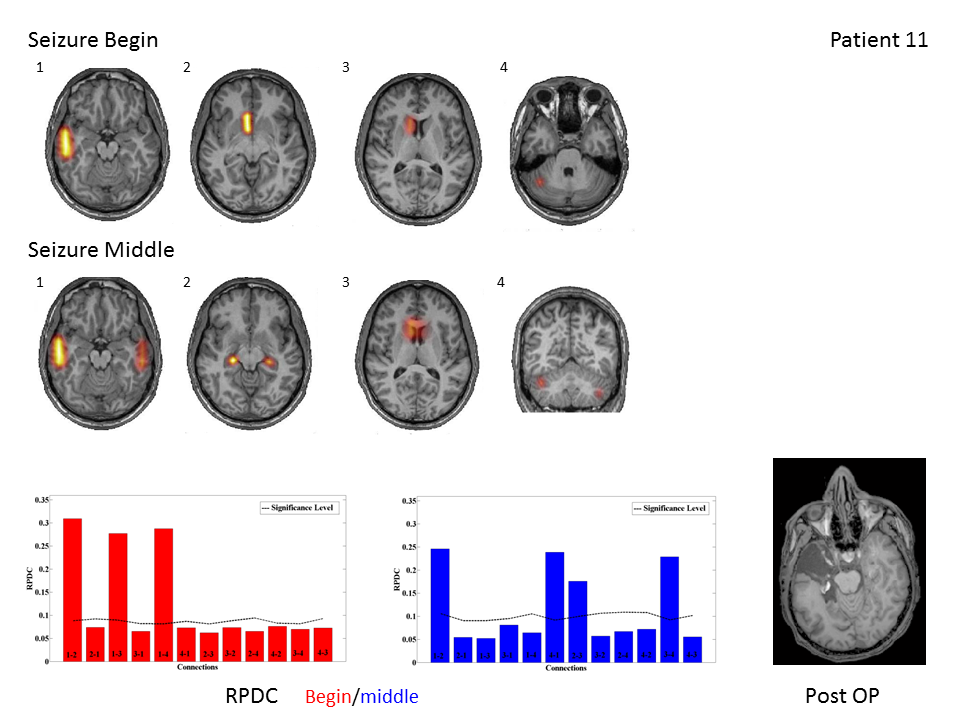

Supplement: Figure S11 — The networks of sources for the seizure begin in the (first row) followed in the (second row) for the seizure middle. The (third row) with the (bar plot in red) shows the RPDC values for the seizure begin and the (blue bar plot) shows the RPDC values for the seizure middle. The postoperative MRI result is shown for patient 11. (TIF) [file pone.0078422.s011.tif]
